# Women Doctors after the War

**Published:** 1915-11-13

**Authors:** 


### WOMEN DOCTORS AFTER THE WAR.

It may, we think, be doubted whether the managers of the London School of Medicine for Women are serving well the cause for which they stand by their reiterated appeal to the public for financial support. The doubt arises solely from the circumstances of the time in which the appeal is made. These circumstances necessarily mean that all appeals for money are scrutinised with a somewhat jealous eye, and that any which are judged to be inopportune and unjustified are apt to excite resentment rather than sympathy. It may be quite true that the School can make out a good case for extension of its buildings, and were the times normal we should have nothing to say in criticism of its appeal. Quite certainly the proposal would command sympathisers, and no one, we imagine, would endeavour to gainsay it. But with funds of immediate importance and of high patriotic value on every hand, and authoritative demands for public and private economy much in evidence, any additional proposed expenditure must expect a doubtful reception.

There are undoubtedly quarters in which the appeal of the school has excited hostile comment. It is pointed out that, at the best, the contribution of women doctors relative to the anticipated deficiency of practitioners can only be a small one, and that none of the new students can possibly be available for practice at an earlier date than five years. By this time—in all probability much earlier—the war will be over, and the men now engaged in military service will have returned to civil practice. In other words, the suggestion is made that to frame a permanent programme on an experience which is admittedly temporary and exceptional is hardly a safe procedure. Again, if, as is stated, the men's schools are largely unoccupied, it surely might be possible to secure some arrangement by which the influx of women students could be accommodated for the time being and until events had lessened the acuteness of the necessity for universal economy. Another consideration which has been advanced is that of all forms of medical education that of women is the one which has the least claim to a subsidy, seeing that in the very nature of things a proportion of the students will forsake the profession for the more congenial joys of domesticity. For our own part we should not allow that all these objections are valid, but the history of the fund, we think, shows that they are widely entertained. Had the School been able to show at a later date that the authorities had made the best of circumstances and had surmounted difficulties by ingenious makeshifts, an appeal for help would have been almost certain to have commanded general sympathy. But in existing circumstances it must be admitted that the institution of a public fund for an ambition which has but a limited motive is not easy to defend, and those responsible for such a step can hardly be surprised if their action is harshly criticised. At the moment it has been reinforced by some distinguished professional authorities; but where the politicians have failed to secure general support we are by no means sure that the professors will succeed. And in any event the cause, which is more important than new buildings, has excited hostility in quarters where this might have been avoided. The objection, we repeat, is not to the appeal in itself but to its presentation when larger and more pressing claims ought to be cultivated.

